# Minimum spanning tree analysis of unimpaired individuals at risk of Alzheimer’s disease

**DOI:** 10.1093/braincomms/fcae283

**Published:** 2024-08-20

**Authors:** Alejandra García-Colomo, David López-Sanz, Cornelis J Stam, Arjan Hillebrand, Martín Carrasco-Gómez, Carlos Spuch, María Comis-Tuche, Fernando Maestú

**Affiliations:** Center for Cognitive and Computational Neuroscience, Complutense University of Madrid, 28223 Pozuelo de Alarcón, Spain; Department of Experimental Psychology, Cognitive Psychology and Speech and Language Therapy, Complutense University of Madrid, 28223 Pozuelo de Alarcón, Spain; Department of Experimental Psychology, Cognitive Psychology and Speech and Language Therapy, Complutense University of Madrid, 28223 Pozuelo de Alarcón, Spain; Department of Clinical Neurophysiology and Magnetoencephalography Center, Amsterdam Neuroscience, Amsterdam UMC, Vrije Universiteit Amsterdam, 1081 HV Amsterdam, The Netherlands; Amsterdam Neuroscience, Brain Imaging, 1081 HV Amsterdam, The Netherlands; Amsterdam Neuroscience, Systems and Network Neurosciences, 1081 HV Amsterdam, The Netherlands; Department of Clinical Neurophysiology and Magnetoencephalography Center, Amsterdam Neuroscience, Amsterdam UMC, Vrije Universiteit Amsterdam, 1081 HV Amsterdam, The Netherlands; Amsterdam Neuroscience, Brain Imaging, 1081 HV Amsterdam, The Netherlands; Amsterdam Neuroscience, Systems and Network Neurosciences, 1081 HV Amsterdam, The Netherlands; Center for Cognitive and Computational Neuroscience, Complutense University of Madrid, 28223 Pozuelo de Alarcón, Spain; Department of Electronic Engineering, Universidad Politécnica de Madrid, 28040 Madrid, Spain; Translational Neuroscience Research Group, Galicia Sur Health Research Institute (IIS-Galicia Sur), SERGAS-UVIGO, CIBERSAM, 36312 Vigo, Spain; Translational Neuroscience Research Group, Galicia Sur Health Research Institute (IIS-Galicia Sur), SERGAS-UVIGO, CIBERSAM, 36312 Vigo, Spain; Center for Cognitive and Computational Neuroscience, Complutense University of Madrid, 28223 Pozuelo de Alarcón, Spain; Department of Experimental Psychology, Cognitive Psychology and Speech and Language Therapy, Complutense University of Madrid, 28223 Pozuelo de Alarcón, Spain; Health Research Institute of the Hospital Clínico San Carlos (IdISSC), 28040 Madrid, Spain

**Keywords:** magnetoencephalography, cognitively unimpaired, minimum spanning tree, p-tau231, brain networks

## Abstract

Identifying early and non-invasive biomarkers to detect individuals in the earliest stages of the Alzheimer’s disease continuum is crucial. As a result, electrophysiology and plasma biomarkers are emerging as great candidates in this pursuit due to their low invasiveness. This is the first magnetoencephalography study to assess the relationship between minimum spanning tree parameters, an alternative to overcome the comparability and thresholding problem issues characteristic of conventional brain network analyses, and plasma phosphorylated tau231 levels in unimpaired individuals, with different risk levels of Alzheimer’s disease. Seventy-six individuals with available magnetoencephalography recordings and phosphorylated tau231 plasma determination were included. The minimum spanning tree for the theta, alpha and beta bands for each subject was obtained, and the leaf fraction, tree hierarchy and diameter were calculated. To study the relationship between these topological parameters and phosphorylated tau231, we performed correlation analyses, for the whole sample and considering the two risk sub-groups separately. Increasing concentrations of phosphorylated tau231 were associated with greater leaf fraction and tree hierarchy values, along with lower diameter values, for the alpha and theta frequency bands. These results emerged for the whole sample and the higher risk group, but not for the lower risk group. Our results indicate that the network topology of cognitively unimpaired individuals with elevated plasma phosphorylated tau231 levels, a marker of Alzheimer’s disease pathology and amyloid-β accumulation, is already altered, shifting towards a more integrated network increasing its vulnerability and hub-dependency, mostly in the alpha band. This is indicated by increases in leaf fraction and tree hierarchy, along with reductions in diameter. These results match the initial trajectory proposed by theoretical models of disease progression and network disruption and suggest that changes in brain function and organization begin early on.

## Teaser

García-Colomo *et al*. show an early topological network reorganization associated with the most incipient increases in the pathology marker p-tau231 in cognitively unimpaired individuals, especially relatives of Alzheimer’s disease. Elevated p-tau231 levels are associated with a star-like network topology.

## Graphical Abstract

**Graphical Abstract:**
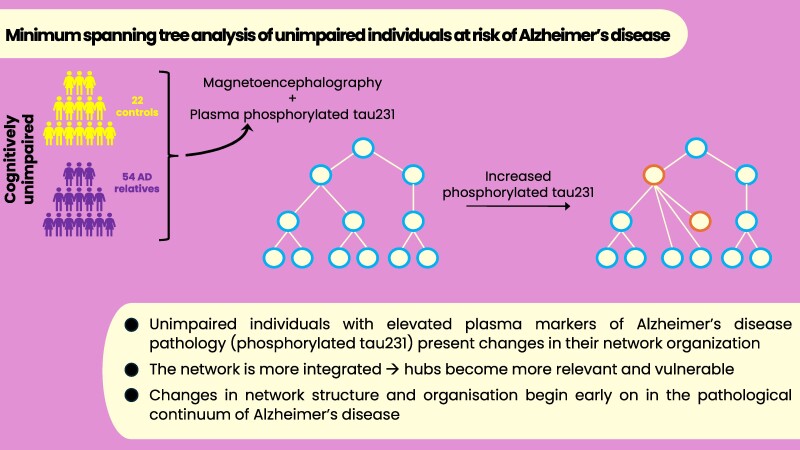

## Introduction

Over the past years, increasing emphasis has been placed on identifying biomarkers that signal the onset of Alzheimer’s disease pathology. Various works have focused on the preclinical phase characterized by the emergence of the earliest alterations with no associated clinical symptom.^1^ Some studies go even further, studying individuals at risk of developing the disease due to their family history or genetic carriage.^2-4^

In this context, electrophysiological measures are being studied as early biomarkers due to their minimal invasiveness and ability to detect incipient functional changes in brain activity.^5,6^ One such biomarker, functional connectivity (FC) has revealed an inverted U-pattern, whereby increases in connectivity during the initial stages are followed by a state of hypoconnectivity.^7-9^ These incipient FC changes are thought to be, at least partially, associated with Aβ accumulation, given the positive feedback loop between Aβ levels and neuronal hyperexcitability that underlies the hyperconnectivity observed in the first stages.^10-12^ In this line, band-specific alterations have been observed associated with Alzheimer’s disease pathology in mild cognitive impairment and Alzheimer’s disease dementia patients,^13,14^ and connectivity alterations have been observed in association with Aβ increases even before Aβ-positivity is observed.^10,12^

To gain a more complete and integrative perspective of FC, the use of graph theoretical analysis has been put forth, considering brain regions as the nodes of the net, and the connectivity value between them the links.^15^ Topological analysis reveals that healthy subjects’ brain networks exhibit attributes such as small-worldness, a hierarchical modular structure, hubness and rich-clubs.^16,17^ Numerous studies have been conducted on the different stages of the Alzheimer’s disease continuum, showing progressive alterations in these topological features. For instance, shifts in small-worldness have been consistently observed across the continuum.^18-21^ Hubs, which are nodes with a high centrality, are also affected throughout the continuum. There is a shift in centrality from posterior areas to anterior ones in the prodromal and dementia stages,^22,23^ and centrality alterations have been reported, although inconsistently, in the preclinical stage in association with incipient Aβ pathology.^24^ It is widely acknowledged that some of these inconsistencies may be, at least partially, due to methodological issues characteristic of conventional brain network analyses that hinder comparability. On the one hand, the choice of a threshold to binarize a connectivity matrix is arbitrary and biases subsequent results since these depend on the density of the network. Similarly, using a surrogate network to normalize against does not fully solve the bias problem either, as differences in average weights influence the network parameters.^23,25^

Minimum spanning tree (MST) analysis constitutes an alternative to solve these methodological issues. The MST of a connectivity matrix with unique weights is a unique sub-graph that connects all the nodes while minimizing the connection weights and avoiding loops. Therefore, unbiased comparison between MST parameters is possible as long as the original matrices are of the same size, i.e. the network consists of the same number of nodes.^16^ Moreover, despite the reduced number of links contained in the MST, several studies have demonstrated that it represents the backbone of information flow in the underlying network, and several MST characteristics are strongly associated with conventional graph theoretical analysis parameters.^26,27^ Lastly, MST analysis has proven capable of detecting clinically relevant changes in the network organization of the brain (for a review on MST articles, see Blomsma *et al*.^28^). In the case of Alzheimer’s disease specifically, several studies have addressed changes towards a more line-like topology of the network, with increases in diameter and reductions in the leaf-fraction in the theta and alpha frequency bands.^6,29,30^ In their study comparing the MST of healthy controls, mild cognitive impairment and Alzheimer’s disease patients, Wang *et al*.^31^ found that MCI patients had a more star-like topology, with a higher leaf fraction (LF) and tree hierarchy (TH),^32^ than Alzheimer’s disease patients, who showed a more line-like one.

Due to their elevated cost, invasiveness and limited accessibility, widely accepted biomarkers (i.e. PET and CSF), are considered poor candidates for early detection and monitoring of disease progression. Therefore, emphasis has been placed on the search for plasma biomarkers.^33^ One such biomarker is the newly discovered species of phosphorylated tau at threonine 231 (p-tau231), which shows abnormal levels in plasma and CSF, as early as the preclinical phase of the continuum. Its levels have been shown to increase parallel to those of Aβ even before Aβ-PET positivity is achieved. As a result of the temporal and disease-specific association between both pathology markers, p-tau231 can be considered a proxy of Aβ pathology, which is linked to early hyperconnectivity.^34-38^

Brain network metrics have the potential to serve as biomarkers for early diagnosis; however, to our knowledge, this is the first study to use MST on a sample of cognitively unimpaired individuals, evaluating the association between MST parameters and p-tau231 levels. Our goal is to disentangle how this plasma pathology marker relates to brain networks and whether these are already altered. We expect to find an association between the MST parameters: LF, diameter and tree-hierarchy and p-tau231 levels, whereby individuals with higher levels of the plasma pathology marker show a shift in their network topology towards a greater dependency on hubs.

## Materials and methods

### Participants

The present study was carried out with a sample of 76 cognitively intact participants with available plasma p-tau231 determinations, extracted from a group of 139 participants with a valid MEG recording. Of these 76, 54 had a direct family history of Alzheimer’s disease (FH+) and 22 had no family history of Alzheimer’s disease or other neurodegenerative disorders (FH−). The present study is part of a larger initiative (‘Study of the anatomo-functional connectome of AD-relatives: an early intervention on cognition and lifestyles’), aimed at tracking participants’ evolution every few years. The main goal of this initiative is to reveal the earliest electrophysiological signs of probable Alzheimer’s disease pathology through the combined use of various neuroimaging, neuropsychological and plasma measurements at different points of the continuum. The data used for the current study belongs to the first follow-up assessment, which is the first evaluation with an available plasma p-tau231 determination.

Every participant underwent a magnetic resonance imaging (MRI) scan during the baseline assessment (which took place 2 to 3 years before the follow-up) that was used for this study. In the follow-up evaluation, all participants underwent an MEG scan, a blood sample extraction for plasma markers determination and a thorough neuropsychological assessment that included the Montreal cognitive Assessment (MOCA) to determine the absence of cognitive impairment (score ≥ 26) and various tests to evaluate the classical cognitive functions: direct and inverse digit span test (Wechsler Memory Scale, WMS-III), phonemic and semantic fluency (controlled oral word association test, COWAT), Boston Naming Test (BNT) and trail making test A and B (TMTA and TMTB). Relevant demographic and clinical information of the sample can be found in Table 1.

**Table 1:** Sample characteristics

	Whole sample	FH+	FH−	Statistic	*P*-value
*N*	76	54	22		
Sex	27(M)/49(F)	17(M)/37(F)	10(M)/12(F)	1.333	0.248
Age	61.382 (6.173)	60.741 (5.779)	62.955 (6.938)	−1.42	0.158
MOCA	28.257 (1.345)	28.308 (1.407)	28.136 (1.207)	0.498	0.619
p-tau231	381.696 (154.983)	390.706 (147.107)	359.58 (174.491)	0.792	0.431
Years of formal education	18.000 (5.149)	17.176 (4.914)	19.647 (5.361)	−1.642	0.107

Values are presented as mean (standard deviation) for the whole sample, relatives’ group (FH+) and controls (FH−). The *t*-tests were used to analyse differences in age, MOCA, p-tau231 and years of education, while *χ*^2^ was used to analyse sex differences. The p-tau231 is presented in pg/ml.

The current study’s exclusion criteria were as follows: (i) history of psychiatric or neurological disorders; (ii) family history of dementia other than Alzheimer’s disease; (iii) presenting a T2-weighted MRI scan with signs of infection, infarction or focal lesions; (iv) consumption of drugs that can influence MEG activity during the past week, alcoholism or chronic use of anxiolytics, neuroleptics, narcotics, anticonvulsants or sedative-hypnotics; (v) being under 50 or above 80 years of age; (vi) a score below 26 in the MOCA; (vii) unusable MEG recording, T1-weighted image or unavailable plasma p-tau321 determination.

This study was approved by the ‘Hospital Clínico San Carlos’ Ethics Committee. All procedures were carried in accordance with internationally accepted guidelines and regulations. Only individuals with a first degree relative (direct descendant or sibling) of a patient diagnosed with confirmed Alzheimer’s disease following the NINCDS-ADRDA criteria^39^ were considered as having a family history.

### Plasma determination

The p-tau231 plasma concentrations were quantified with a competitive enzyme-linked immunosorbent assay (MyBiosource, Inc., USA, MBS724296), following the manufacturers’ instructions. Duplicates for each sample were performed. The optical density was measured at 450 nm using an automated microplate reader (Biochrom ASYS UVM 340, Cambridge, UK) and Mikrowin 2000 software (Berthold Technologies, Germany). Values are expressed as pg/ml.

### MEG recordings and preprocessing

A 306-sensor Vectorview MEG system (Elekta AB, Stockholm, Sweden) at the Center for Biomedical Technology (Madrid, Spain) was used to record 4 min of eyes-closed resting state brain activity of each participant. Head movements during the recording were tracked with four coils, two placed on the forehead and two on the mastoids. Coils’ position and each participant’s head shape were digitized with a Fastrack Polhemus system (Polhemus, Colchester, VT, USA). Two sets of bipolar electrodes were used to record electrooculographic and electrocardiographic activity. Recordings were carried out inside a magnetically shielded room and participants were instructed to remain still and relax. Data were acquired with a sampling rate of 1000 Hz, using an online filter between 0.1 and 330 Hz. In order to remove the contribution of head movements and distant sources outside the brain, the signal was processed with the temporal expansion of the signal space separation method, implemented by Neuromag Software (MaxFilter version 2.2, correlation limit 0.90, time window 10 s).^40^

Next, MEG signal was preprocessed. First, physiological and jump artefacts were removed by an electrophysiology expert with Fieldtrip.^41^ We then used an ICA-based algorithm to remove EOG and EKG components from the. The data were divided into segments of four consecutive seconds (4096 samples) of artefact-free activity. Thirty segments were randomly selected for each subject and kept for further analyses. Subsequent analyses only used data from magnetometers due to the redundant nature of the data after applying the signal space separation method.^42^

### Source reconstruction

Source-space MEG signal was reconstructed using each individual’s T1 brain MRI. Acquisition of MRI recordings was done with a general electric 1.5 T system equipped with high-resolution antenna and a homogenization phased array uniformity enhancement filter (fast spoiled gradient echo sequence, TR/TE/TI = 11.2/4.2/450 ms; flip angle 12°; slice thickness 1 mm, 256 × 256 matrix and FOV 25 cm).

The source model was created with a regular grid composed of 4560 sources (equivalent current dipoles), separated from each other by 1 cm forming a cube. Reconstruction and further analyses only considered sources corresponding to cortical regions as indicated by the automated anatomical labelling atlas, resulting in a total of 1210 brain sources. Next, this source model was linearly transformed into each participant’s individual T1-weighted image. SPM12 brain segmentation^43^ was employed to create single-shell head models.^44^ The head model, the source model information and a modified spherical solution were combined to calculate the lead field matrix.

Sensor space signals were filtered between 2 and 45 Hz using a 450th-order finite input response filter designed with a Hann window. We employed a two-pass filtering approach to prevent phase distortion. Additionally, we added 2000 samples of real data padding at the beginning and end of the signal to avoid edge effects. The 1210 sources were grouped into 80 cortical areas according to the AAL atlas. Only the source closest to the centroid for each area was considered further. We used LCMV beamformer as an inverse model, which we calculated using Fieldtrip with the leadfield and the epoch-averaged covariance matrix with a regularization of 1%. The source-space centroids’ time series were obtained by multiplying the weights marked by the filter and the sensor-space time series of each sensor in each epoch.

### FC analysis

Source-space FC analyses were carried out using the open-access software BrainWave (version 1.2.15; https://github.com/CornelisStam/BrainWave.git). FC between regions was estimated using the amplitude envelope correlation with leakage correction (AEC-c) for all 30 epochs of each subject (for an in-depth explanation of the method, see Schoonhoven *et al*.,^45^). A band-pass filter was applied to the centroids’ time-series in the theta (4–8 Hz), alpha (8–13 Hz) and beta (13–30 Hz) frequency bands that are known to show a more robust signal-to-noise ratio during human electrophysiological recordings.^46^

While the amplitude envelope correlation calculates the linear correlation of the envelopes signals after a band-pass filter and Hilbert transformation have been applied,^47^ the AEC-c removes the zero-lag correlations that could be the result of spurious signal coupling due to volume conduction (i.e. signal leakage in source-space). To do this, a symmetric pairwise orthogonalization of the signal is performed before the calculation of the AEC.^48^ Multiple studies have shown the reliability of AEC-c as a metric of FC.^45,48^

As a result, an 80 by 80 AEC-c adjacency matrix that contains the FC values between all pairs of centroids is obtained for each epoch of each subject in the three frequency bands under study.

### MST analyses

A separate MST was constructed for every epoch in each of the three frequency bands under study using the reciprocal AEC-c adjacency matrix by applying Kruskal's algorithm^49^ in order to obtain the backbone of the network. This algorithm rearranges the links in an ascending order and starts building the MST adding, first, the link with the strongest weight and then the following smallest one until all nodes (N) are connected in a loopless sub-graph with N-1 links. If adding one link generates a cycle, said link is discarded. In our study, the resulting sub-graphs contained 80 nodes and 79 links.

Global MST parameters were calculated for every MST generated: LF, diameter (D) and TH, which provide topological properties of the tree. In a tree, the number of nodes that are connected only to one other node is referred to as the number of leaves (*L*). In a line-like tree, *L* is only 2 (the two nodes at the beginning and the end of the line are leave nodes), whereas in a star-like tree, there are N-1 leaf nodes (all nodes are leaves, except the one central node that is connected to all others). The LF is a measure of centrality and is defined as *L*, divided by the maximum number of possible leaves, which coincides with the number of edges (*m*).


(1) LF=L/m

The diameter is a measure of network efficiency that refers to the largest distance (*d*), as the number of links, between any two nodes of the tree, normalized by the total number of links. In a line-like tree, diameter will be equal to the number of edges, while a star-like tree diameter will be 2. This implies a tight association between diameter and *L*, whereby the largest possible diameter will decrease with an increasing number of *L*.^16^


(2) D=d/m

The optimal configuration of a tree should balance efficiency, which is expressed by a small diameter value, while preventing hub overload. The measure TH reflects this trade-off.


(3) $$\mathrm{TH}=L/(2mBC_{\max})$$

BC_max_ refers to the maximum value of betweenness centrality in the MST, and the betweenness centrality for each node is calculated as the portion of shortest paths that go through it. TH ranges between 0 and 1, describing a non-linear network topology change across its range. In a line-like tree, TH would approach 0, whereas for a star-like tree, TH would approach 0.5. Trees with intermediate leaf numbers between these extreme topologies will have greater TH values. It is hypothesized that a value of TH that approaches 1 is indicative of a network topology that optimizes a trade-off between integration and segregation is hypothesized.^32^

### Statistical analysis

Statistical analyses were carried out in Matlab R2019b. First, the relationship between p-tau231 and demographic variables known to impact AD pathology was addressed. A Pearson correlation analysis was used to evaluate the relationship between p-tau231 and age and years of education, while a *t*-test was used to check for differences in p-tau231 levels between males and females.

First, each subject’s LF, diameter and TH values from all 30 epochs were averaged for each frequency band under study. This left us with one single value for each of the topological parameters in the theta, alpha and beta bands for each subject (see Supplementary Table 1 for the MST parameters’ values of the whole sample, FH+ and FH−).

Next, we studied whether the three topological properties from the MST were associated with the p-tau231 levels of each participant across our whole sample. For this, we conducted a Pearson correlation analysis. A false discovery rate test with a *q* = 0.1 threshold was applied to account for multiple comparisons.

Next, to study whether Alzheimer’s disease relatives (FH+) and controls (FH−) showed different patterns of association between their topological properties and p-tau231 we performed correlation analyses for these two groups separately. Once more, a false discovery rate test was applied to account for multiple comparisons, considering the results from both the FH+ and FH− groups in all metrics and bands together.

The aforementioned non-linear nature of the TH makes the interpretation of its values for trees with non-extreme topologies difficult, as a deviation from 1 may be indicative of a shift towards either of the extremes. To aid interpretation, we performed a Pearson correlation between the TH and LF of FH+ and FH− only for significant TH results.

## Results

No significant differences could be observed in p-tau231 levels neither between FH+ and FH− (*t* = 0.792; *P* = 0.431) nor between sexes (*t* = −0.980; *P* = 0.329). Likewise, no significant correlation was found between p-tau231 and age (*ρ* = 0.122; *P* = 0.295) or years of education (*ρ* = 0.044; *P* = 0.761).

**Figure 1:**
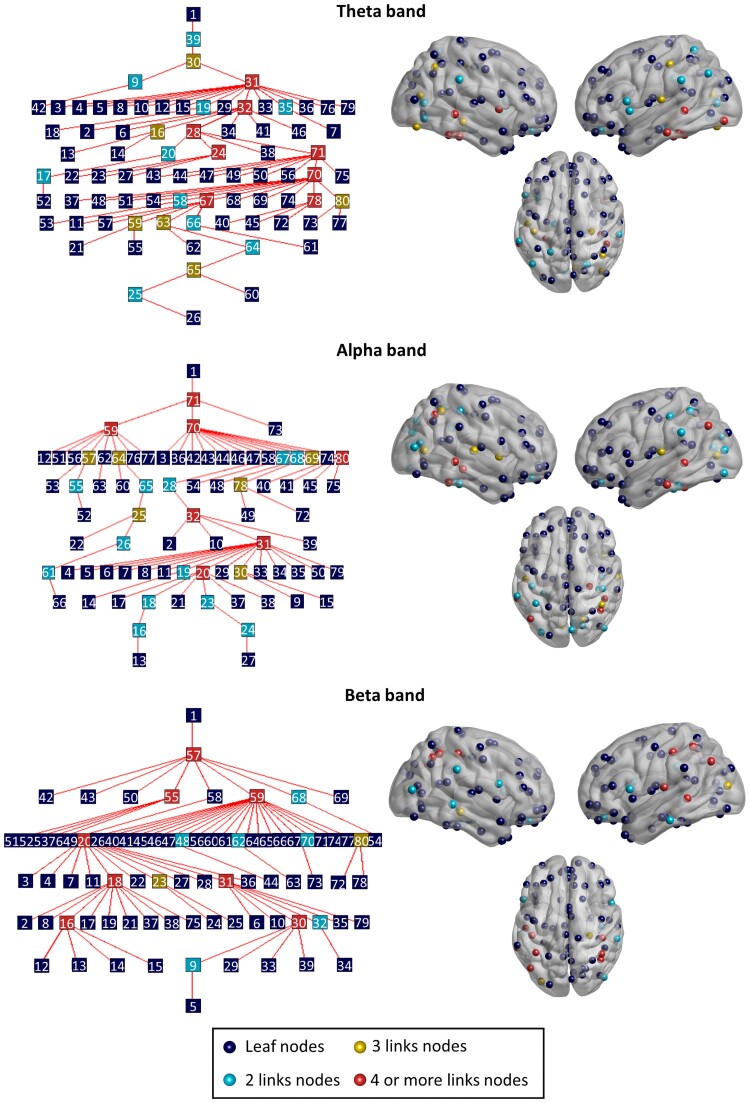
**MST depictions and centroid distribution.** To the left, the resulting MSTs for the theta, alpha and beta frequency bands for the whole sample. On the right-hand side, the localization of each of the centroids in the cortex coloured according to their degree.

A qualitative inspection of the resulting MSTs (Fig. 1) revealed that some of the nodes with higher degrees were consistent across bands (for the correspondence between numbers and the complete list of regions, see Supplementary Table 2). Regions such as the bilateral inferior and middle temporal gyri, the left superior temporal gyrus and left angular gyrus were, at least, in two of the three measured frequency bands among the nodes with four or more links. The MSTs for all frequency bands had a similar degree structure, showing a relatively similar number of nodes in the tree with four or more links (eight for theta and beta; seven for alpha). Likewise, the right hippocampus had three links in both theta and beta bands. On the other topological extreme, leaf nodes were also relatively consistent, showing around 60 nodes (53–63 depending on the frequency range) with only one connection and distributed across similar brain regions.

**Figure 2:**
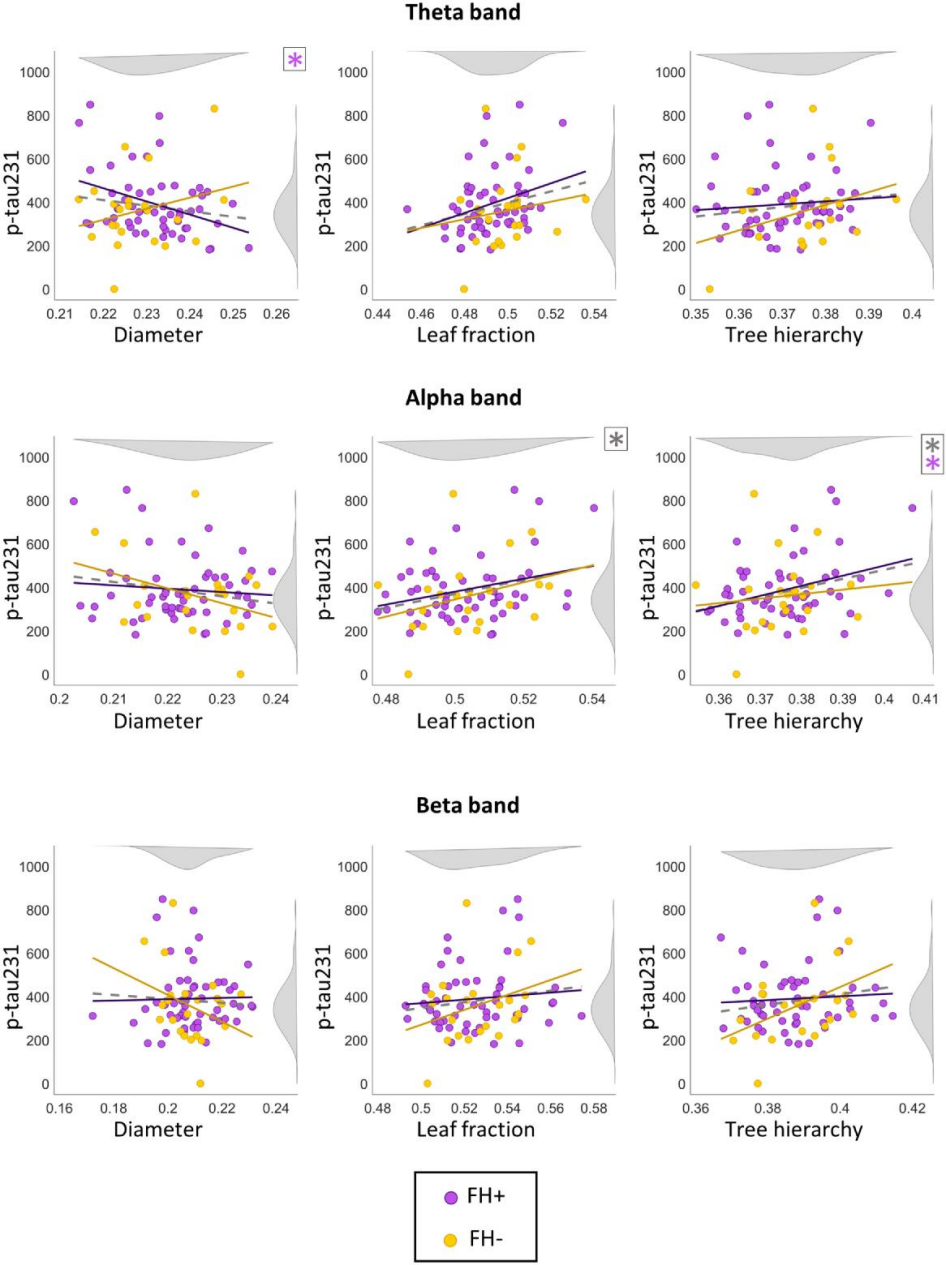
**Scatter plots showing the relationship between p-tau231 and the MST parameters: diameter, LF and TH, for the theta, alpha and beta frequency bands, in the different groups.** The regression for the whole sample is indicated with a grey dashed line. Asterisks are coloured according to which group shows a significant Pearson correlation between the parameter and p-tau231. Relatives of AD patients (FH+), non-relatives (FH−). The dashed line represents the whole sample. Significance is established after false discovery rate correction and results can be found in Table 2.

When studying the whole sample, p-tau231 showed significant positive correlations (see Table 2) that survive FDR (the threshold for significance was 0.016; Fig. 2) with the LF (*ρ* = 0.291; *P* = 0.011; adj-*q* = 0.071) and the TH in the alpha band (*ρ* = 0.276; *P* = 0.015; adj-*q* = 0.071). The LF in the theta band (*ρ* = 0.227; *P* = 0.048; adj-*q* = 0.144) showed a tendency towards significance but did not survive FDR correction.

**Table 2:** Relationship between MST parameters and plasma p-tau231 values

	Whole sample		FH+		FH−		
	*ρ*	*P* (adj-q)	*ρ*	*P* (adj-q)	*ρ*	*P* (adj-q)	FH+ *ρ* versus FH− *ρ P*
Theta band							
LF	0.227	0.048 (0.144)	0.308	0.023 (0.140)	0.152	0.498 (0.625)	0.269
Diameter	−0.15	0.194 (0.250)	−0.373	0.005* (0.098)	0.258	0.246 (0.401)	0.007
TH	0.139	0.232 (0.261)	0.089	0.521 (0.626)	0.325	0.140 (0.309)	0.178
Alpha band							
LF	0.291	0.011* (0.071)	0.289	0.034 (0.152)	0.307	0.164 (0.309)	0.471
Diameter	−0.188	0.103 (0.232)	−0.091	0.514 (0.625)	−0.352	0.108 (0.309)	0.152
TH	0.276	0.015* (0.071)	0.344	0.011* (0.098)	0.105	0.641 (0.678)	0.173
Beta band							
LF	0.154	0.184 (0.250)	0.104	0.455 (0.625)	0.302	0.172 (0.309)	0.220
Diameter	−0.062	0.591 (0.591)	0.023	0.868 (0.867)	−0.317	0.149 (0.309)	0.096
TH	0.169	0.142 (0.250)	0.067	0.631 (0.678)	0.428	0.047 (0.168)	0.073

Values are presented as Pearson’s *ρ* estimated value for the whole sample, relatives’ group (FH+) and control group (FH−). The *P*-values are reported as the raw value and, in parenthesis, the adjusted *q*-value after FDR. *Significance after FDR testing. For the whole sample, the threshold value to consider significance was 0.016, for the sub-samples the threshold was 0.011.

When looking at the FH+ and FH− groups (Fig. 2), the FH+ showed significant positive correlations between most MST topological properties and p-tau231 (Table 2). A significant negative correlation was found between p-tau231 and the diameter in the theta band (*ρ* = −0.373; *P* = 0.005; adj-q = 0.098). On the other hand, a positive correlation was found with the TH in the alpha band (*ρ* = 0.344; *P* = 0.011; adj-*q* = 0.098). The LF in theta band (*ρ* = 0.308; *P* = 0.023; adj-*q* = 0.140) and alpha (*ρ* = 0.289; *P* = 0.034; adj-*q* = 0.152) showed a trend towards significance but did not survive FDR correction. Likewise, the FH− group only showed a trend towards significance with the TH (*ρ* = 0.428; *P* = 0.047; adj-*q* = 0.168) in beta, which did not survive FDR correction. The FDR threshold to consider significance was 0.011.

Lastly, a significant positive correlation was found between the TH and LF of FH+ individuals in the alpha band (*ρ* = 0.836; *P* < 0.001).

## Discussion

To the best of our knowledge, this is the first effort to address the relationship between plasma p-tau231, an early, easily accessible marker of Alzheimer’s disease pathology, and MEG network topology in cognitively unimpaired individuals. Our results show a relationship between the levels of p-tau231 and topological alterations that are maintained or emerge when studying the higher-risk sub-group of Alzheimer’s disease relatives. This underscores the potential of topological metrics and MST parameters as biomarkers for identifying early alterations associated with Alzheimer’s disease plasma pathology markers, as well as an early and accessible measure to evaluate the influence of potential disease-modifying interventions due to its sensitivity to subtle changes.

Plasma p-tau231 has become pivotal for AD’s preclinical stage as it mirrors Aβ species’ increases, predicts elevated Aβ before Aβ-PET positivity is achieved and is discriminant of the different stages.^34,37,38,50^ Consequently, in the present study, p-tau231 has been used as a proxy for Aβ and Alzheimer’s disease pathology. We found a positive correlation between p-tau231 and the MST parameters LF and three hierarchies, and a negative correlation with diameter. These results suggest that higher levels of this pathology marker in cognitively unimpaired individuals are already associated with a more star-like brain network topology, indicative of increased randomness.^28^ Interestingly, studies performed in later stages of the continuum have consistently reported a shift towards a line-like topology, which constitutes another indicator of the decentralization and disconnection characteristic of Alzheimer’s disease.^6,29,31^ Studies performed in the intermediate state of mild cognitive impairment yield inconsistent results^30,31,51^ that could reflect an inflexion point, where the star-like topology, characteristic of the initial stages, shifts towards a line-like topology, characteristic of later stages of the continuum.

Our results reflect an increase in network randomness among unimpaired individuals with increasing p-tau231 levels that is opposite to the one observed among Alzheimer’s disease patients.^6,29,31^ Notably, this pattern would mirror the changes described in FC and neuronal excitability as Alzheimer’s disease pathology emerges and evolves. The initial increase in excitability and connectivity tied to the incipient Aβ pathology later leads to a state of hypoexcitability, associated with tau accumulation and neuronal death.^7,9,11,12^ Similarly, a previous study from our group^52^ described an initial longitudinal increase in connectivity in key regions for Alzheimer’s disease among first-degree relatives, who also showed a positive correlation between their FC values and p-tau231. Altogether, our finding of an initial increase in network randomness may constitute a network-scale manifestation of the early Aβ and excitability increases, prior to any cognitive symptom, as congruently suggested by its association with p-tau231 increases. Future evaluations of these individuals should include classically accepted markers of pathology to corroborate this hypothesis.

Different metrics contribute distinct nuances to the interpretation of the results. The positive correlation between p-tau231 and LF indicates an increased dependency upon hubs in the alpha band. Numerous studies have been conducted addressing alterations in this frequency band for its sensitivity to Alzheimer’s disease pathology throughout the continuum, which evidences power reductions,^53,54^ peak slowing^53,55,56^ and connectivity alterations.^57^ Moreover, previous studies have also found alterations in LF in Alzheimer’s disease patients within this frequency band.^6,29^ However, these, along with fMRI and conventional graph theory centrality studies performed on the dementia stage contrast with our current findings. Peraza *et al*.^6^ and Wang *et al*.^31^ found reduced LF among Alzheimer’s disease patients, while Yu *et al*.^58^ reported a loss of centrality in posterior hubs. This initial rise in hub relevance, as demonstrated by the current LF results, is thought to lead to a hub overload, followed by a decline in centrality further along the continuum. This aligns with the theoretical model proposed by Stam,^16^ according to which hub vulnerability is characteristic of Alzheimer’s disease and follows an inverted U-shape pattern. Initially, hubs start receiving information directly from lower-level nodes of the hierarchy. While this reorganization results in an initial increase of hub centrality, if sustained, leads to hub overload and a subsequent centrality loss. Furthermore, evidence from computational models and experimental studies suggests that hub vulnerability can be activity-dependent and the result of neuronal hyperexcitability, which, as aforementioned, is tied to early Aβ increases.^12,16,59^ Consequently, the observed increase in LF in the alpha frequency band tied to higher levels of p-tau231 among healthy individuals may constitute the earliest manifestation of topological reorganization that would subsequently give way to a loss of LF and centrality alterations.

Regarding diameter results, FH+ individuals with higher p-tau231 levels showed a reduction in this parameter in the theta band, therefore exhibiting a greater efficiency in information flow due to the reduction in the largest distance between any two nodes.^28^ This finding in the theta band is congruent with previous literature that highlights its relevance in the disease continuum, showing consistent increases in power,^53^ enhanced synchrony^60^ and even alterations in diameter in Alzheimer’s disease patients.^6^ However, in contrast with our results, the dementia stage is typically associated with an increased diameter.^29,30^ Classical graph analyses performed within this same stage of the continuum using path length (i.e. average shortest path between any two nodes), which positively correlates with diameter,^26^ report similar although less consistent results. Increases in path length among Alzheimer’s disease patients have been interpreted as a sign of segregation due to connectivity loss, and a shift from a small world-like network architecture.^18,61,62^ Although our result indicates a reduction in diameter with elevated p-tau231 levels, theoretical studies pose that the network diameter is inversely proportional to the number of leaves in a graph^51^; therefore, this result is consistent with our findings showing increases in LF in association with p-tau231, as a network with more leaves and star-like topology will exhibit a smaller distance between nodes, contributing to a greater efficiency and the aforementioned increase in hub dependency and overload. Furthermore, reductions in diameter and efficiency increases are also expected in association with increases in connectivity, which have been reported in cognitively unimpaired subjects with a family history of Alzheimer’s disease and associated with p-tau231.^52^ Altogether, these results point towards a deviation from the optimal network topology, which is known to underlie cognitive alterations observed in the prodromal and dementia stages of the continuum^63^ as well as with the risk of conversion to dementia.^64^

Lastly, we studied the relationship between plasma p-tau231 levels and TH values, which characterize the hierarchical topology of a tree, quantifying the relationship between integration and hub overload, taking values close to 0 for line-like trees, to 0.5 for star-like ones, and approaches 1 when an optimal balance between integration and hub overload is achieved.^16^ Our results showed a positive relationship between TH in the alpha band and p-tau231 levels. Given the non-linear behaviour of this measure,^16^ we performed additional analyses to interpret this result. TH values were positively associated with LF in our sample, suggesting a more star-like topology in relation to increases in TH. This tendency is indicative of a greater demand on network hubs. This is congruent with our LF results for the alpha band, which also point towards a more star-like topology among individuals with elevated p-tau231 levels, confirming a greater dependency upon hubs. As aforementioned and in line with theoretical and experimental evidence, we hypothesize that individuals who eventually progress in the Alzheimer’s disease continuum will later show the opposite topology after hubs become overloaded due to pathology progression. Wang *et al*.^31^ reported an increase in TH for MCI participants and a subsequent decline in Alzheimer’s disease in comparison to controls; showing that after a stage of hub overload, the network transitions to a state of disconnection. Lastly, the positive correlations are maintained only when looking at the FH+ sub-group. Since having a family history of Alzheimer’s disease represents a compound risk factor,^65^ it is possible that these individuals have additional unknown characteristics that make them more prone to pathological changes. Notwithstanding, considering the absence of significant rho differences between sub-groups and the reduced FH− sample size, which can impact statistical power, future studies should enrich this sub-group and validate these results.

This pioneering study addresses the relationship between the plasma pathology marker p-tau231 and different MST parameters among cognitively unimpaired individuals. However, this study is not without shortcomings. Future assessment rounds should include other established techniques alongside plasma biomarkers to improve individuals’ risk classification and improve these novel markers’ validity. Still, plasma biomarkers constitute a more ecological, minimally invasive, cost-efficient and readily accessible alternative. As previously mentioned, the sample size could represent a limiting factor, especially in the case of the FH− sub-group, as it can have an impact on statistical power. Importantly, this study is part of a longitudinal initiative aimed at tracking these individuals’ evolution for decades and the next evaluation round, which has already been approved, includes cohort enrichment plans. Lastly, future studies should take a node-level approach to complement global network topological findings. Still, upon a qualitative inspection of the MSTs obtained for the theta, alpha and beta bands, nodes with higher degree are regions commonly regarded as hubs, which is in agreement with the notion that the MST represents the backbone of the network.

Taken together, the present findings indicate that the network topology of cognitively unimpaired individuals with elevated plasma p-tau231 levels, a marker of AD and Aβ pathology, shows alterations in line with previous experimental and theoretical models, and the described changes associated with the early stages of the continuum. The observed shift towards a more star-like or integrated but vulnerable and hub-dependent network, mostly in the alpha band, is remarkable for various reasons. First, it evidences network changes that match the initial trajectory proposed by theoretical models of disease progression and network disruption, highlighting MEG sensitivity to subtle changes in network topology associated with incipient plasma pathology markers. Second, it provides the first demonstration of the relationship between network topology and the novel plasma marker p-tau231. Therefore, this study expands previous knowledge and suggests that changes in brain function and organization emerge along with incipient pathological markers. Moreover, the techniques employed in this study are minimally invasive, a key strength for future protocols of early detection and disease tracking.

## Supplementary Material

fcae283_Supplementary_Data

## Data Availability

The data that support the findings of this study are available from the corresponding author, upon reasonable request. BrainWave software is available online at (https://github.com/CornelisStam/BrainWave.git) and the remaining scripts to perform statistical analysis can be found as Supplementary material.
