# Supplementary material for: Minimum spanning tree analysis of unimpaired individuals at risk of Alzheimer’s disease
Source: Brain Commun. 2024 Aug 20;6(5):fcae283. doi: 10.1093/braincomms/fcae283 (PMC11369931; doi:10.1093/braincomms/fcae283)
Supplement: fcae283_Supplementary_Data [file fcae283_supplementary_data.zip › Supplementary_tables.docx]

**Supplementary Table 1.** MST parameters’ values in the theta, alpha and beta bands for the whole sample, relatives’ group (FH+) and control group (FH-). A Student’s t-test was performed to compare the FH+ and FH- groups. None of the comparisons survive FDR correction at q=0.1.

|  | Whole sample Mean(+-std) | FH+ Mean(+-std) | FH- Mean(+-std) | p value |
| --- | --- | --- | --- | --- |
| Theta band | | | | |
| Leaf fraction | 0.494 (0.014) | 0.492 (0.013) | 0.499 (0.013) | 0.039 |
| Diameter | 0.231 (0.009) | 0.232 (0.009) | 0.228 (0.009) | 0.074 |
| Tree hierarchy | 0.372 (0.009) | 0.37(0.009) | 0.375 (0.009) | 0.05 |
| Alpha band | | | | |
| Leaf fraction | 0.504 (0.014) | 0.503 (0.014) | 0.504 (0.014) | 0.935 |
| Diameter | 0.223 (0.008) | 0.222 (0.008) | 0.225 (0.009) | 0.166 |
| Tree hierarchy | 0.376 (0.010) | 0.376 (0.011) | 0.375 (0.009) | 0.724 |
| Beta band | | | | |
| Leaf fraction | 0.526 (0.018) | 0.525 (0.019) | 0.526 (0.015) | 0.907 |
| Diameter | 0.209 (0.011) | 0.209 (0.011) | 0.208 (0.009) | 0.669 |
| Tree hierarchy | 0.388 (0.011) | 0.387 (0.011) | 0.388 (0.010) | 0.791 |

**Supplementary Table 2**. List of AAL regions with their full name and identification numbers

| **Id** | **Name of the region** | **Id** | **Name of the region** |
| --- | --- | --- | --- |
| 1 | Left Gyrus Rectus | 41 | Right Olfactory cortex |
| 2 | Left Olfactory cortex | 42 | Right Superior Frontal gyrus, Orbital |
| 3 | Left Superior Frontal gyrus, Orbital | 43 | Right Superior Frontal gyrus, Medial Orbital |
| 4 | Left Superior Frontal gyrus, Medial Orbital | 44 | Right Middle Frontal gyrus, Orbital |
| 5 | Left Middle Frontal gyrus, Orbital | 45 | Right Inferior Frontal gyrus, Orbital |
| 6 | Left Inferior Frontal gyrus, Orbital | 46 | Right Superior Frontal gyrus |
| 7 | Left Superior Frontal gyrus | 47 | Right Middle Frontal gyrus |
| 8 | Left Middle Frontal gyrus | 48 | Right Inferior Frontal gyrus, Opercular |
| 9 | Left Inferior Frontal gyrus, Opercular | 49 | Right Inferior Frontal gyrus, Triangular |
| 10 | Left Inferior Frontal gyrus, Triangular | 50 | Right Superior Frontal gyrus, Medial |
| 11 | Left Superior Frontal gyrus, Medial | 51 | Right Supplementary Motor area |
| 12 | Left Supplementary Motor area | 52 | Right Paracentral lobule |
| 13 | Left Paracentral lobule | 53 | Right Precentral gyrus |
| 14 | Left Precentral gyrus | 54 | Right Rolandic operculum |
| 15 | Left Rolandic operculum | 55 | Right Postcentral gyrus |
| 16 | Left Postcentral gyrus | 56 | Right Superior Parietal gyrus |
| 17 | Left Superior Parietal gyrus | 57 | Right Inferior Parietal gyrus |
| 18 | Left Inferior Parietal gyrus | 58 | Right Supramarginal gyrus |
| 19 | Left Supramarginal gyrus | 59 | Right Angular gyrus |
| 20 | Left Angular gyrus | 60 | Right Precuneus |
| 21 | Left Precuneus | 61 | Right Superior Occipital lobe |
| 22 | Left Superior Occipital lobe | 62 | Right Middle Occipital lobe |
| 23 | Left Middle Occipital lobe | 63 | Right Inferior Occipital lobe |
| 24 | Left Inferior Occipital lobe | 64 | Right Calcarine fissure and surrounding cortex |
| 25 | Left Calcarine fissure and surrounding cortex | 65 | Right Cuneus |
| 26 | Left Cuneus | 66 | Right Lingual gyrus |
| 27 | Left Lingual gyrus | 67 | Right Fusiform gyrus |
| 28 | Left Fusiform gyrus | 68 | Right Heschls gyrus |
| 29 | Left Heschls gyrus | 69 | Right Superior Temporal gyrus |
| 30 | Left Superior Temporal gyrus | 70 | Right Middle temporal gyrus |
| 31 | Left Middle temporal gyrus | 71 | Right Inferior Temporal gyrus |
| 32 | Left Inferior Temporal gyrus | 72 | Right Temporal pole, Superior Temporal gyrus |
| 33 | Left Temporal pole, Superior Temporal gyrus | 73 | Right Temporal pole, Middle temporal gyrus |
| 34 | Left Temporal pole, Middle temporal gyrus | 74 | Right Parahippocampus |
| 35 | Left Parahippocampus | 75 | Right Cingulate gyrus, Anterior part |
| 36 | Left Cingulate gyrus, Anterior part | 76 | Right Cingulate gyrus, Middle part |
| 37 | Left Cingulate gyrus, Middle part | 77 | Right Cingulate gyrus, Posterior part |
| 38 | Left Cingulate gyrus, Posterior part | 78 | Right Insula |
| 39 | Left Insula | 79 | Left Hippocampus |
| 40 | Right Gyrus Rectus | 80 | Right Hippocampus |
